# HIV-1 Tat: Its Dependence on Host Factors is Crystal Clear

**DOI:** 10.3390/v2102226

**Published:** 2010-10-06

**Authors:** Iván D’Orso, Alan D. Frankel

**Affiliations:** Department of Biochemistry and Biophysics, University of California, 600 16th Street, San Francisco, CA 94158-2280, USA; E-Mail: ivan.dorso@ucsf.edu

**Keywords:** HIV-1, Tat, transcription, P-TEFb, retroviruses

## Abstract

HIV-1 transcription is regulated at the level of elongation by the viral Tat protein together with the cellular elongation factor P-TEFb, which is composed of cyclin T1 and Cdk9 subunits. The crystal structure of a Tat:P-TEFb complex (Tahirov, T.H.; Babayeva, N.D.; Varzavand, K.; Cooper, J.J.; Sedore, S.C.; and Price, D.H. Crystal structure of HIV-1 Tat complexed with human P-TEFb. *Nature* **2010**, *465*, 747–751.) reveals molecular details of Tat and its interactions that have eluded investigators for more than two decades and provides provocative insights into the mechanism of Tat activation.

HIV-1, like many viruses, co-opts the cellular machinery to orchestrate its life cycle, including its transcriptional program, and the recent crystal structure of a viral-host protein complex has revealed new facets of this interplay [1]. In this case, the virally encoded Tat protein recruits the host factor positive transcription elongation factor b (P-TEFb) to an RNA hairpin formed at the 5’-end of nascent viral RNAs (TAR) to activate a switch to transcription elongation [2]. P-TEFb is a cyclin-dependent kinase complex composed of Cdk9 and cyclin T1 (CycT1) subunits that serves key roles in globally regulating the elongation phase of RNA polymerase II (RNAP II) transcription [2,3]. The discovery of P-TEFb as an essential Tat cofactor [4,5], and the finding that CycT1 binds to TAR RNA cooperatively with Tat [6,7], provided a basic understanding of Tat mechanism. Tat and P-TEFb are recruited to TAR, where Cdk9 phosphorylates the elongation-related factor DSIF as well as Ser2 residues in the C-terminal domain (CTD) repeats of RNAP II to generate the processive IIo form of the enzyme [2]. P-TEFb also exists in a catalytically inactive form bound to the 7SK snRNP, which is composed of 7SK snRNA and the Larp7, Mepce, and Hexim1 or Hexim2 proteins [2,8–10]. Tat promotes dissociation of the 7SK snRNP to activate Cdk9 [11–13], and recent evidence suggests that the inhibited complex together with Tat are recruited to the HIV-1 promoter during initiation and that Tat and P-TEFb are later transferred to TAR on the nascent RNA, thereby releasing the inhibitory 7SK snRNP [13]. Despite this progress on the molecular mechanism of Tat activation, the structure of Tat and complexes with its interacting partners has remained elusive. The NMR structure of TAR RNA bound to a small RNA-binding peptide from Tat has been known for some time [14–16], but no structural details about the Tat activation domain or its relationship to the transcriptional machinery had been revealed. That is, until the recent high-resolution crystal structure of the Tat:P-TEFb complex from Tahirov, Price, and co-workers [1]. This *tour-de-force* study, which relied on painstaking purification of the complex co-expressed from baculovirus vectors, provides deep insights into the assembly of this viral-host protein complex and helps explain how Tat subverts P-TEFb to regulate HIV-1 transcription. This work follows on the heels of a previous breakthrough crystal structure of P-TEFb, which showed plasticity of the cyclinT1-Cdk9 interface and the importance of Cdk9 phosphorylation for activation of the kinase and substrate recognition [17].

Tat is comprised of two exons. The first exon is sufficient for viral transcription and contains an activation domain (AD; residues 1–48), RNA-binding domain (RBD; residues 49–57), and a C-terminal extension (residues 58–72) (Figure 1). The second exon (residues 73–86 or 73–101, depending on the isolate) does not have a primary role in viral transcription but may have other functions during viral replication [18]. In the 2.1 Å crystal structure reported by Tahirov *et al*., the Tat AD is found to acquire a relatively extended conformation upon interaction with P-TEFb (Figure 1), forming an extensive interface with both CycT1 (88% of the covered Tat surface) and Cdk9 (12% of the covered surface) (Figure 2), with a total buried surface area twice the average for a stable protein-protein interaction [19]. The cysteine-rich portion of the Tat AD is folded into a compact structure with two α-helices that coordinate two Zn ions. It is clear from the structure that the Tat AD by itself does not adopt a stable fold, and its extended conformation in some ways resembles ribosomal proteins that fold only in the context of the rRNA scaffold [20]. The Tat RBD and C-terminal extension (residues 50–86) are not observed in the structure, probably because the complex lacks TAR, which is believed to help define an as yet unknown conformation of the arginine-rich RBD [14–16].

Earlier biochemical data demonstrated that the Tat AD coordinates two Zn ions through cysteine-rich zinc finger (ZnF)-like motifs and that Cys261 in CycT1 forms a Zn-mediated bridge to Tat (Figure 2) [7,21]. The precise type of metal coordination remained unclear because the ZnF motifs did not correspond to any particular consensus sequence, but the structure now shows that one Zn ion is tetrahedrally coordinated by Cys22, His33, Cys34, and Cys37 of Tat and the second by Cys25, Cys27, and Cys30 of Tat and most likely Cys261 of CycT1 (Figure 1). No density was observed for residues 253–266 of CycT1, which corresponds to the previously defined Tat:TAR recognition motif (TRM) important for RNA binding [7] (dashed line, Figure 2), and a broadened density was observed for Cys261, consistent with biochemical data suggesting this region has high mobility or intrinsic disorder [22]. It remains possible that a different residue from a disordered part of Tat occupies the position ascribed to Cys261. Nonetheless, the observed coordination patterns neatly correspond to earlier mutagenesis and binding studies of Tat indicating the importance of these particular residues in Zn-dependent TAR binding and Tat activation [7,23]. Many human transcription factors contain cysteine- or histidine-containing ZnFs that typically fold into modular domains that recognize DNA or RNA or mediate protein-protein interactions [24,25]. The Zn domains in Tat bear no structural similarity to the known classes of ZnFs or other metalloproteins, and the reliance on CycT1 to complete the metal coordination indicates how dependent Tat structure is on its host partner. Moreover, the complex displays a large number of intermolecular hydrogen bonds between Tat and P-TEFb compared to few intramolecular contacts within Tat alone, further implying that Tat folding is primarily determined by the P-TEFb interface.

One can envisage at least two practical advantages for a protein, particularly from a small virus, to evolve such a strong dependence on interacting partners to adopt structure: First, an intrinsically disordered or structurally flexible protein may adapt to more than one binding partner and facilitate competing interactions that may be required in a temporal manner for function [26,27]. For example, Tat may engage proteins other than P-TEFb during transcription initiation and the transition into elongation, including other host elongation factors or 7SK snRNP components [13,28,29]. Second, a virus with a very limited genome, such as HIV-1, can optimize its coding capacity by evolving small proteins that do not possess extensive polypeptide chains needed to stabilize a protein fold. Rather, they can rely on existing structural scaffolds provided by the host, provided that they evolve a sufficiently tight and specific protein-protein interface. The use of Zn domains to help fold portions of Tat also is economical, as exogenous metals provide a good way to stabilize small protein modules [30]. HIV-1 also appears to have evolved other strategies to make economical use of its coding capacity. For example, the small viral protein Rev uses an adaptable binding surface to recognize multiple sites on the RRE RNA and form a large homo-oligomeric assembly that exports viral RNAs from the nucleus to the cytoplasm [31].

Another possible advantage to structural flexibility is the ability of a virus to populate large areas of sequence space that include functionally permissive mutations. Indeed, Tat tolerates up to 40% sequence variation without noticeable loss of transcriptional activity [32]. For the conserved residues, the Tahirov *et al*. structure is satisfyingly consistent with many observed evolutionary constraints. Most obviously, the amino acids that confer tetrahedral geometry for metal coordination are fully conserved, as are amino acids in the protein-protein interface where mutations would cause clashes with P-TEFb. The need for other conserved positions is not yet explained. For example, position 2 is always a negatively charged amino acid (Asp or Glu), position 26 is a highly conserved aromatic residue (Tyr or Phe), and position 28 is a highly conserved Lys. Lys28 is acetylated (although not in the structure) and modulates the formation of Tat:P-TEFb complexes on TAR [33]. Acetylation of Lys28, located on the second ZnF, might help stabilize the Tat:P-TEFb protein-protein interface or participate in remodeling when bound to TAR. Indeed, it has been proposed that recognition of the apical loop of TAR, which requires both Tat and CycT1, may involve conformational changes to one or the other protein that consequently facilitate RNA recognition [6,7]. Such questions, and also the requirements of other conserved residues, highlight the importance of visualizing Tat:P-TEFb complexes with RNA and possibly other Tat-host protein complexes. One previous study determined the structure of a related equine infectious anemia virus (EIAV) Tat-CycT1 fusion protein bound to EIAV TAR [34] and found that both Tat and CycT1 contact the RNA hairpin, with the EIAV Tat RBD adopting a helical conformation and flanking regions contributing to assembly of the ternary complex. Strong similarities between the ZnFs and core regions of Tat suggest that the CycT1 interaction surface is the most conserved structural feature, whereas differences between the TARs and RBDs of HIV-1 and EIAV do not allow the HIV-1 interaction to be modeled. It will be interesting to determine if HIV-1 Tat utilizes similar principles of RNA recognition, where residues outside the RBD also coordinate the assembly of the RNA-protein complex.

Tahirov *et al*. reported the structure not of just one Tat:P-TEFb complex but of two complexes, with and without a bound ATP analog [1]. The ATP-bound structure permits comparison with a previously reported P-TEFb structure [17] and suggests that Tat may remodel the complex and possibly explain how it regulates Cdk9 activity. The interface between CycT1 and Cdk9 involves a relatively small surface [1,17]. Tat inserts itself into a groove at the heterodimer interface, augmenting the surface and possibly creating a more stable and active P-TEFb complex. This insertion leads to some interesting conformational rearrangements. First, Tat unfolds and disorders a small α-helix in CycT1 that is part of the TRM [7], thus exposing a buried surface of CycT1 that allows recognition of the ZnF and adjacent regions of Tat. The TRM is required for TAR loop recognition, raising the possibility that the additional exposed surfaces also might be positioned to contact the RNA as the nascent transcript emerges from RNAP II. Second, the conformational changes in CycT1 cause an 8.5° rotation in the position of the Cdk9 subunit and shifts the positions of a phosphorylated Thr loop (T-loop) in Cdk9 (Figure 2), an autophosphorylation site that promotes kinase activity [17], and two other loops near the ATP-binding site. These concerted rearrangements may help activate P-TEFb and modify the substrate specificity of Cdk9, possibly staging phosphorylation so that Ser5 of the RNAP II CTD becomes modified first in paused transcription complexes and Ser2 becomes modified later as TAR emerges and the transition into productive elongation complexes ensues [35,36]. One caveat to comparing Tat-bound P-TEFb to unbound P-TEFb is that CycT1 in the unbound structure [17] contained three mutations (Q77R, E96G, and F241L), where E96 is located close to the Tat and Cdk9 interfaces such that loss of contacts to neighboring arginines potentially could explain some of the conformational differences ascribed to Tat binding. This caveat notwithstanding, it is tempting to speculate that some of the proposed Cdk9 rearrangements induced by Tat, or changes in kinase activity, also may lead to release of the 7SK snRNP, based on how Hexim1 of the 7SK snRNP is believed to inhibit P-TEFb and analogous to how p27^kip1^ inhibits Cdk2:cyclinA [1]. Further structural and functional studies will be needed to illuminate how the inhibitory 7SK snRNP complex is assembled into Tat complexes [13,29], disassembled by Tat [11,12], or ejected upon TAR binding [13,37].

The structure by Tahirov *et al*. represents a major milestone in Tat and HIV-1 biology and, like all landmark papers, raises a host of interesting new questions. Some of these will be addressed by additional structural studies, most immediately of complexes with TAR. While some is known about how Tat interacts with the bulge region of TAR, the structural basis for TAR recognition by the full Tat:P-TEFb complex, including the TAR loop, remains incomplete. It is possible that the interactions observed or conformational transitions implied by the present structure will differ in the context of RNA or other factors present in transcription initiation or elongation complexes. Some of these may be important for Cdk9 activation or substrate recognition. How the Tat:P-TEFb complex interacts with or is mutually exclusive with the inhibitory 7SK snRNP complex remains to be explored. Novel discoveries are still emerging that suggest molecular mimicry between viral and host components, such as TAR and 7SK snRNA [38], and finding differences between these host and viral-host complexes may reveal unique surfaces for targeted drug design. The structural basis for how posttranslational modifications of Tat, including Lys acetylation and Arg methylation, affect complex formation and activity is not understood. And the broader implications of the structure for Tat inhibitor design remain for the future. Obvious targets seem to be regions of P-TEFb that interact with Tat but are not used for its normal cellular function, which still must be defined. The Tat:P-TEFb structure provides a wonderful starting point for deeper studies of the mechanism of Tat activation, inhibition, and control of cellular transcription elongation. Once again, viral structural biology provides a window into host cell biology.

## Figures and Tables

**Figure 1:**
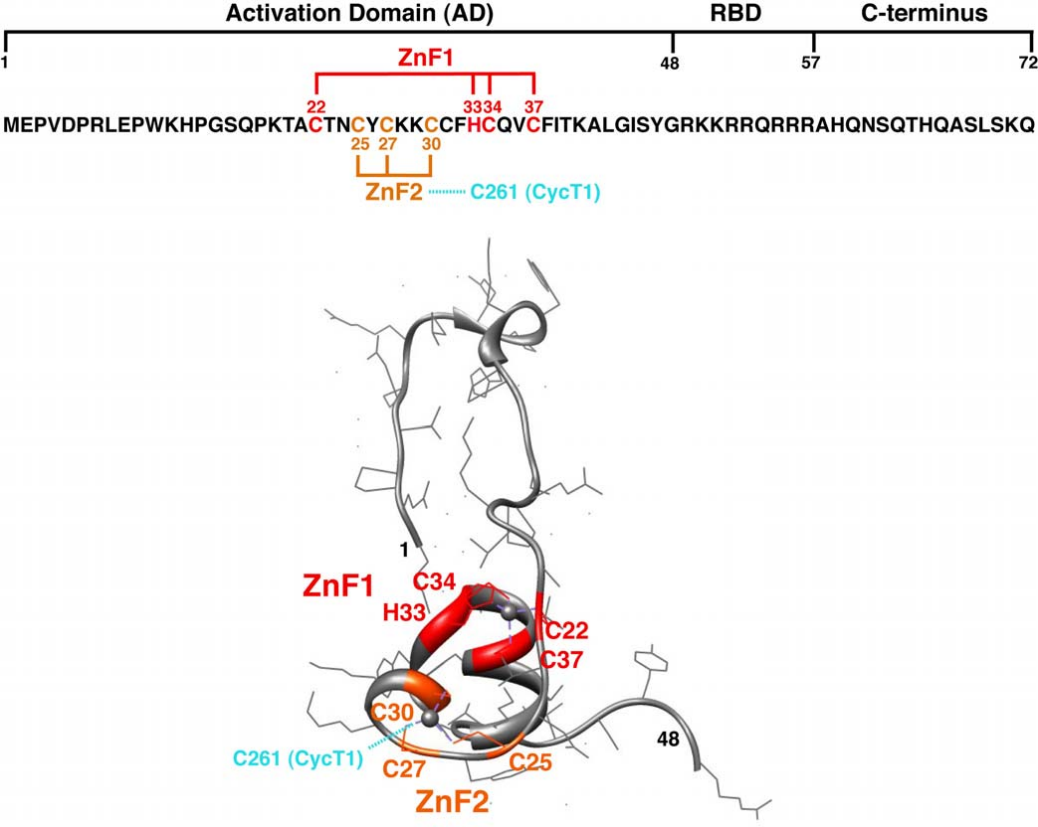
Sequence of Tat and structure of the Tat activation domain (AD) in the context of the Tat:P-TEFb crystal structure. The sequence of Tat exon 1 (residues 1–72) is shown along with its different modules: AD (residues 1–48), RBD (residues 49–57) and C-terminus (residues 58–72). Within the AD, the two Zinc-finger motifs, ZnF1 and ZnF2, are shown in red and orange. The metal coordination of ZnF2 is completed by C261 of CycT1 (cyan). Only the Tat AD is observed in the structure and becomes folded upon the P-TEFb interaction (chain C in PDB 3MI9). The structure representations shown in Figures 1 and 2 were generated using Chimera software (UCSF).

**Figure 2:**
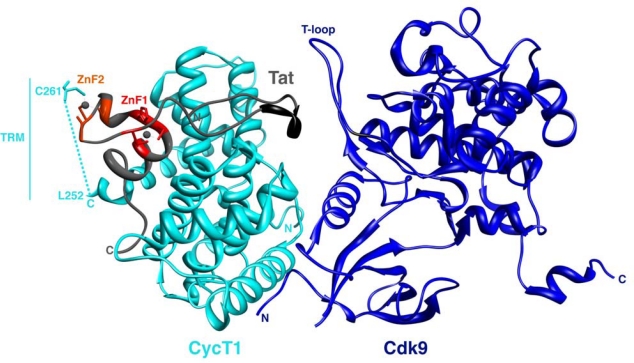
Structure of the Tat:P-TEFb complex. The Tat AD (gray) is observed to interact primarily with CycT1 (cyan) but also with the Cdk9 subunit (blue). The dashed line represents the distance between the last residue in CycT1 that is ordered in the structure (L252) and C261 (PDB 3MI9). TRM denotes the Tat-TAR Recognition Motif in CycT1 [7] (residues 250–262) that is disordered in the Tat:P-TEFb complex.

## References

[1] Tahirov TH, Babayeva ND, Varzavand K, Cooper JJ, Sedore SC, Price DH (2010). Crystal structure of HIV-1 Tat complexed with human P-TEFb. Nature.

[2] Peterlin BM, Price DH (2006). Controlling the Elongation Phase of Transcription with P-TEFb. Mol Cell.

[3] Bres V, Yoh SM, Jones KA (2008). The multi-tasking P-TEFb complex. Curr Opin Cell Biol.

[4] Mancebo HS, Lee G, Flygare J, Tomassini J, Luu P, Zhu Y, Peng J, Blau C, Hazuda D, Price D, Flores O (1997). P-TEFb kinase is required for HIV Tat transcriptional activation *in vivo* and *in vitro*. Genes Dev.

[5] Zhu Y, Pe'ery T, Peng J, Ramanathan Y, Marshall N, Marshall T, Amendt B, Mathews MB, Price DH (1997). Transcription elongation factor P-TEFb is required for HIV-1 tat transactivation *in vitro*. Genes Dev.

[6] Wei P, Garber ME, Fang SM, Fischer WH, Jones KA (1998). A novel CDK9-associated C-type cyclin interacts directly with HIV-1 Tat and mediates its high-affinity, loop-specific binding to TAR RNA. Cell.

[7] Garber ME, Wei P, KewalRamani VN, Mayall TP, Herrmann CH, Rice AP, Littman DR, Jones KA (1998). The interaction between HIV-1 Tat and human cyclin T1 requires zinc and a critical cysteine residue that is not conserved in the murine CycT1 protein. Genes Dev.

[8] Zhou Q, Yik JH (2006). The Yin and Yang of P-TEFb regulation: implications for human immunodeficiency virus gene expression and global control of cell growth and differentiation. Microbiol Mol Biol Rev.

[9] Jeronimo C, Forget D, Bouchard A, Li Q, Chua G, Poitras C, Therien C, Bergeron D, Bourassa S, Greenblatt J, Chabot B, Poirier GG, Hughes TR, Blanchette M, Price DH, Coulombe B (2007). Systematic analysis of the protein interaction network for the human transcription machinery reveals the identity of the 7SK capping enzyme. Mol Cell.

[10] Krueger BJ, Jeronimo C, Roy BB, Bouchard A, Barrandon C, Byers SA, Searcey CE, Cooper JJ, Bensaude O, Cohen EA, Coulombe B, Price DH (2008). LARP7 is a stable component of the 7SK snRNP while P-TEFb, HEXIM1 and hnRNP A1 are reversibly associated. Nucleic Acids Res.

[11] Barboric M, Yik JH, Czudnochowski N, Yang Z, Chen R, Contreras X, Geyer M, Matija Peterlin B, Zhou Q (2007). Tat competes with HEXIM1 to increase the active pool of P-TEFb for HIV-1 transcription. Nucleic Acids Res.

[12] Sedore SC, Byers SA, Biglione S, Price JP, Maury WJ, Price DH (2007). Manipulation of P-TEFb control machinery by HIV: recruitment of P-TEFb from the large form by Tat and binding of HEXIM1 to TAR. Nucleic Acids Res.

[13] D'Orso I, Frankel AD (2010). RNA-mediated displacement of an inhibitory snRNP complex activates transcription elongation. Nat Struct Mol Biol.

[14] Puglisi JD, Tan R, Calnan BJ, Frankel AD, Williamson JR (1992). Conformation of the TAR RNA-arginine complex by NMR spectroscopy. Science.

[15] Aboul-ela F, Karn J, Varani G (1995). The structure of the human immunodeficiency virus type-1 TAR RNA reveals principles of RNA recognition by Tat protein. J Mol Biol.

[16] Long KS, Crothers DM (1999). Characterization of the solution conformations of unbound and Tat peptide-bound forms of HIV-1 TAR RNA. Biochemistry.

[17] Baumli S, Lolli G, Lowe ED, Troiani S, Rusconi L, Bullock AN, Debreczeni JE, Knapp S, Johnson LN (2008). The structure of P-TEFb (CDK9/cyclin T1), its complex with flavopiridol and regulation by phosphorylation. EMBO J.

[18] Romani B, Engelbrecht S, Glashoff RH (2010). Functions of Tat: the versatile protein of human immunodeficiency virus type 1. J Gen Virol.

[19] Janin J (1997). Specific *versus* non-specific contacts in protein crystals. Nat Struct Biol.

[20] Ban N, Nissen P, Hansen J, Moore PB, Steitz TA (2000). The complete atomic structure of the large ribosomal subunit at 2.4 A resolution. Science.

[21] Frankel AD, Bredt DS, Pabo CO (1988). Tat protein from human immunodeficiency virus forms a metal-linked dimer. Science.

[22] Das C, Edgcomb SP, Peteranderl R, Chen L, Frankel AD (2004). Evidence for conformational flexibility in the Tat-TAR recognition motif of cyclin T1. Virology.

[23] Rice AP, Carlotti F (1990). Mutational analysis of the conserved cysteine-rich region of the human immunodeficiency virus type 1 Tat protein. J Virol.

[24] Patikoglou G, Burley SK (1997). Eukaryotic transcription factor-DNA complexes. Annu Rev Biophys Biomol Struct.

[25] Klug A (2010). The discovery of zinc fingers and their applications in gene regulation and genome manipulation. Annu Rev Biochem.

[26] Dunker AK, Silman I, Uversky VN, Sussman JL (2008). Function and structure of inherently disordered proteins. Curr Opin Struct Biol.

[27] Nobeli I, Favia AD, Thornton JM (2009). Protein promiscuity and its implications for biotechnology. Nat Biotechnol.

[28] He N, Liu M, Hsu J, Xue Y, Chou S, Burlingame A, Krogan NJ, Alber T, Zhou Q (2010). HIV-1 Tat and host AFF4 recruit two transcription elongation factors into a bifunctional complex for coordinated activation of HIV-1 transcription. Mol Cell.

[29] Sobhian B, Laguette N, Yatim A, Nakamura M, Levy Y, Kiernan R, Benkirane M (2010). HIV-1 Tat assembles a multifunctional transcription elongation complex and stably associates with the 7SK snRNP. Mol Cell.

[30] Frankel AD, Berg JM, Pabo CO (1987). Metal-dependent folding of a single zinc finger from transcription factor IIIA. Proc Natl Acad Sci U S A.

[31] Daugherty MD, D'Orso I, Frankel AD (2008). A solution to limited genomic capacity: using adaptable binding surfaces to assemble the functional HIV Rev oligomer on RNA. Mol Cell.

[32] Campbell GR, Loret EP (2009). What does the structure-function relationship of the HIV-1 Tat protein teach us about developing an AIDS vaccine. Retrovirology.

[33] D'Orso I, Frankel AD (2009). Tat acetylation modulates assembly of a viral-host RNA-protein transcription complex. Proc Natl Acad Sci U S A.

[34] Anand K, Schulte A, Vogel-Bachmayr K, Scheffzek K, Geyer M (2008). Structural insights into the cyclin T1-Tat-TAR RNA transcription activation complex from EIAV. Nat Struct Mol Biol.

[35] Zhou M, Halanski MA, Radonovich MF, Kashanchi F, Peng J, Price DH, Brady JN (2000). Tat modifies the activity of CDK9 to phosphorylate serine 5 of the RNA polymerase II carboxyl-terminal domain during human immunodeficiency virus type 1 transcription. Mol Cell Biol.

[36] Buratowski S (2009). Progression through the RNA polymerase II CTD cycle. Mol Cell.

[37] Barboric M, Lenasi T (2010). Kick-sTARting HIV-1 transcription elongation by 7SK snRNP deporTATion. Nat Struct Mol Biol.

[38] Durney MA, D'Souza VM (2010). Preformed Protein Binding Motifs in 7SK snRNA: Structural and Thermodynamic Comparisons with Retroviral TAR. J Mol Biol.

